# Sexual Exhaustion (Neurasthenia)

**Published:** 1880-06

**Authors:** George M. Beard

**Affiliations:** Fellow of the New York Academy of Medicine; Vice-President of the American Academy of Medicine; Member of the American Neurological Association, etc., etc.


VOL. I.——*June, 1880.*——No. 6.

ARTICLE I. PART II.

### SYMPTOMS OF SEXUAL EXHAUSTION (SEXUAL NEURASTHENIA.)

BY GEORGE M. BEARD, A. M., M. D.

Fellow of the New York Academy of Medicine; Vice-President of the American Academy of Medicine; Member of the American Neurological Association, etc., etc.

*Exhaustion after Defecation and Urination.*—Where the prostate is especially irritable, it is observed that the operations of defecation and urination are sometimes followed by a feeling of exhaustion, which may last for some minutes or several hours, according to circumstances.

This exhaustion is of a reflex character, the irritation of the urine or the faeces acting upon the prostate and the movement of the muscles required in the acts of defecation and urination, together constituting irritation which re-acts on the whole nervous system. It is for this reason that patients of this class sometimes feel very well until after they have had a passage from the bowels. To these patients a slight diarrhœa is very weakening.

*General or Reflex Symptoms.*—The general or reflex symptoms of neurasthenia are of a reflex or sympathetic character, and are of great interest scientifically without regard to their practical relations.

Increase of reflex actions is one of the most instructive and interesting of all the results of the nervous sensitiveness of modern times; the higher and more delicate the organization the more numerous and more complex and original the reflexes in disease; in the fine organization the effects of any local irritation are both more easily transferred from one part of the body to another and are more quickly and sensitively felt. Success in diagnosing and treating all functional diseases of the nervous system is measured by the thoroughness with which we search for sources of reflex irritation. The first thing in studying any cases of this kind is to find out from what part of the body they are reflected; only in a minority of cases are the symptoms and the disease exclusively confined to the same locality; where reflex irritation is not the sole factor in exciting the symptoms it is an important and not to be overlooked complication, and one which, if neglected, will be sufficiently powerful to keep us from curing our patient.

Some of the following symptoms are found in cases where there is no reason to suspect any disorder of the reproductive apparatus; but rarely if ever do we find all these symptoms or any considerable number of them united in a single case unless some sexual disturbance is the starting point or accompaniment.

*Irresistible Masturbation.*—There is a state to which the term sexual inebriety might be applied, the feature of which is involuntary or at least irresistible masturbation. Those who are afflicted with this symptom can no more restrain themselves than can the inebriate or the opium eater. The passion sometimes comes upon these sufferers suddenly, almost instantaneously, and with a force that at once, like the incoming of a flood, carries all away before it—resolutions, modesty and even fear of immediate pain. The authentic cases of this are most remarkable, and are of the highest psychological value.

One of my medical friends informs me that he had under his care a gentleman who is attacked suddenly and without special warning in the street, and with such fury that he must and does at once betake himself to some hiding place and find relief in masturbation; the call is as imperious as that for urination or defecation.

Dr. Hyde of Chicago has reported a case of a young man who left the table while eating, hastened to his room and was there detected indulging furiously.

Two or three cases I have known who masturbated in sleep unconsciously, or at least involuntarily. Niemeyer and some other writers assert that in all or nearly all cases when the symptoms of sexual exhaustion are complained of the habit of masturbation is still maintained; this however is an assumption that is not justified by facts.

*Palmer Hyperidrosis,* (*Sweating of the Hands.*)—Cold and clammy perspiration in the palms of the hands is almost, if not absolutely, diagnostic of difficulty with the reproductive apparatus. This condition is found, not in young men alone, but in the married—indeed in all who have been especially excessive, or in any way have brought on a state of sexual neurasthenia.

While it is entirely possible that this symptom might exist in a person who had no other sign of sexual disturbance I have yet to see a case of that kind. Even in females this hand sweating is almost always associated with uterine disease.

No symptom is more demonstrably and speedily under the direct influence of the nervous system than this; in a moment the hands may become dry or moist and may change from one condition to the other. Although the palms of the hands are much the more likely to be affected the whole hand in some cases becomes moist so as to quickly wear out a pair of gloves, and to be a constant embarrassment; the excitement of meeting and being introduced to a stranger brings on this symptom so that before one is ready to shake hands they will be in full perspiration.

The feet in some cases are affected equally with the hands; the symptoms coming and going according to the state of the system and the presence or absence of excitement.

This symptom resembles blushing in the inability of the sufferer to control it and in this, that it becomes worse the more one attempts to control it.

My studies of this subject have convinced me that the palmer hyperidrosis like everything else in the constitution is hereditary—running in families in a number of cases; it may follow one even after marriage, but as is the case with all this family of symptoms, the tendency is to grow milder with years. Sweating of the scrotum is likewise to be noted, accompanied, it may be, by a disagreeable odor. This local sweating may follow any special excess and often accompanies varicocele. Sometimes this scrotal hyperidrosis is confined to the left side where also varicocele is most frequent. Night sweats also appear in sexual exhaustion and cause the sufferer to apprehend consumption.

*Prostration and Nervousness after Coition.*—Nervousness and sleeplessness and headache and prostration following coition, when performed but rarely, are evidences of some form of sexual exhaustion and are worthy of attention even though there should be no other sign of trouble in that function. In practice we find, however, that this symptom does not exist alone; it is always a part of a group.

In one extraordinary case for which I was consulted coition as infrequent as once in three months produced unpleasant effects, but the individual thus sensitive could sleep at any time and as long as he wished and was capable of severe and responsible mental labor.

There are cases not so uncommon as was formerly supposed, whose genital function is in a condition analagous to the extreme forms of nervous dyspepsia—intolerant of any functional activity, no matter how mild or cautious.

There are persons who cannot eat even a crust of bread without experiencing discomfort; just so there are persons who cannot indulge at all in coition without experiencing more or less of the symptoms that are supposed to follow only great excess; this function is a poison to them—the only difference in the evil effects of moderation and excess being of degree only.

Sexual intercourse in the normal man is a sedative and tonic; it induces sleep, quickens the appetite and inspires activity; the slight and pleasing languor that follows it are physiological. In cases of this kind, as I have many times noticed, coitus is followed the next day by an unusual sensitiveness of the urethra, as is observed on introducing the sound or other instruments.

In the time of their appearance these bad effects of sexual excitation are remarkably inconsistent; in some persons not manifesting themselves for two days, or the second day, in other cases appearing at once after the act. This same inconsistency obtains in other symptoms and after the exercise of other functions; thus a clergyman who consulted me was troubled not by the blue Monday, but by a blue Tuesday—the re-action from the labors of Sabbath and delaying for forty-eight hours. Likewise the phenomena of indigestion sometimes follow at once after a meal; in other cases not until several hours.

*Lumbar and Groin Pain.*—Investigations that have been made in the symptom called oxaluria have brought out the fact that lumbar and groin pain very often accompany great excess of oxalates and urates in the urine; and a still closer analysis of the whole subject of neurasthenia shows that lumbar pain is a very frequent attendant indeed of the sexual variety of that disorder.

This pain is sometimes accompanied with tenderness of some of the lumbar vertebrae, sometimes not; in some instances there is pain at the very tip of the spine as in women, and quite often there is a sense of aching and heaviness in the hips and groins. In cases where the irritability is very profound, pain in the tip of the spine or lumbar region comes on at once after coition or even after any excitement.

A vague and very hard to-be-defined nervousness often goes with this pain and aching, extending down the limbs and over the whole body. Patients sometimes speak of nervousness of the lower limbs as an immediate and regular sequence of coition—they say it is not exactly pain, but a fidgety feeling.

*Neurasthenic Voice.*—The peculiar soft and uncertain voice to which elsewhere I have applied the term atonic or neurasthenic voice, in men is usually connected with some form of genital difficulty, and recovers in proportion as there is improvement in the vigor of the reproductive system. I do not say that it is impossible for one to have this peculiar voice without any genital complication; but if there be such cases they must be exceptional.

Debility of all kinds, the exhaustion of fever and convalescence, are revealed in this voice weakness; but this special quality or voice here described is quite different, and can be well recognized by those who are familiar with it, this voice is not only weak; it is soft, doubtful, uncertain, timid as though the patient feared to speak. The patient speaks as the heart sometimes beats in the condition called irritable heart, that is, as though each moment it were on the point of stopping. Such voices soon tire in long conversation or in public speech.

*Morbid fears.*—Morbid fears as I have a number of times described them, are found in so many phases of neurasthenia in both sexes, that they cannot be said to be diagnostic of reproductive weakness; and yet they are often found in persons who are not all right in the reproductive system. There is often a history of natural or unnatural excess which perhaps may have been abandoned long before, while the sequel appears in these morbid fears. This is one of the symptoms that must be taken in connection with other and associated symptoms before a diagnosis is made. Anthropophobia or fear of society with peculiar aversion of the eyes, is almost diagnostic of sexual trouble in the male.

*Injection of the conjunctiva, coming and going.*—Cases of this kind often look as if they had been on a spree. They may look so in the morning and in the afternoon be all natural in the eyes. One time I congratulated one of my patients on the improved appearance of the conjunctiva. He replied, in two hours my eyes may look as bad as ever. It is quite probable that many organs of the body are in the same vascular condition as the eyes in these and other forms of nervous exhaustion. This vascular congestion is not necessarily attended with any asthenopia or dimness of vision.

*Certain forms of Asthenopia.*—That there is a very intimate relation indeed between the genital function and the eyes, is now well established, and in some instances attention must first be given to the cause before we can remove the effect. Muscular insufficiency is often, but not always found to be the pathological accompaniment of this pain and aching of the eyes, and a fullness of the veins of the retina. Glasses alone, no matter how wisely selected, are not sufficient in these cases.

Not a few of these cases complain of attacks of dimness of vision. In several cases that had previously consulted ophthalmotologists, or that have been referred to them by me, nothing abnormal could be detected.

*Excessive, causeless and protracted flushing of face and neck.*—Blushing is physiological or constitutional. Flushings as a pathological symptom is of considerable interest. It is experienced in those who have no appreciable disorder of the reproductive apparatus; but it is certainly most annoying and most varied in its phases in those who are troubled somewhat in that direction. It is certain that in these cases flushing is more likely to be permanent.

In some cases mechanical excitation of the genital apparatus excites flushing even when there is no feeling or suggestion of shame; the symptom being physical purely. I have now under my care a patient who when a sound is passed flushes between the eyes and on portions of the nose, and there is a patch of redness of the size of half a dollar on the lower portion of the right cheek; but the rest of the cheek is not affected. The parts that are flushed are very warm to the touch. This patient was not aware of this peculiarity until I called his attention to it. It is very interesting to see how quickly this symptom gives way to treatment directed to the cause.

In the case of a physician now under my observation, this flushing persists during an afternoon or evening, when in company. This person is also troubled with excessive sweating of the hands and feet. In his case there is not so much sexual exhaustion as sexual irritability; the result in part of the nervous diathesis, and in part of prolonged continence.

*Pain in the vertex.*—In women, pain in the vertex has been looked upon as pretty clearly indicating disorder in the reproductive system, and with just reason. I have seen severe pain in that region of the head relieved instantaneously by an application of faradic electricity to the neck of the womb.

In men, this special phase of head pain is more rare; but when it exists it is as a result or attendant of some disturbance of the prostate gland, or of some part of the complex genital machinery. Instead of positive pain in the vertex there may be a feeling of heat and burning as is often felt in the spine in spinal irritation.

*Irritable heart.*—The condition now called irritable heart is analogous to irritable uterus, irritable ovary, irritable breast, irritable testes, irritable eye and ear, spinal irritation and cerebral irritation; and it is very often associated with the nervous dyspepsia of an irritable stomach; indeed nearly all if not all the organs of the body and the whole body may fall into a state of irritability with symptoms that simulate and suggest organic disease, and which are very often mistaken for organic disease.

In the irritable heart of the sexually exhausted there may be the extreme of slowness or the extreme of rapidity, from thirty or forty up to one hundred and ten and thirty. Slight exertion or trifling emotional influence suffice to put up the pulse all the way from twenty-five to seventy-five per cent.; climbing mountains, going up hills even, or but one or two flights of stairs, the sudden meeting of a friend, a start or shock of an exceedingly insignificant nature are enough to produce this effect; it is very hard to convince such persons that they have not organic disease of the heart, especially as precordial pain and uneasiness and distress and intermitting go with this high or low pulse.

Dr. Fothergill in his work on Diseases of the Heart has described this condition, very truthfully though not fully, and suggests also its frequent dependence on the genital function.

*Defective memory and mental control.*—The statement so often made by patients that memory has declined as a result of their nervous exhaustion, are not credited by the profession; but in this observation the patients are often though not always in the right. Memory rightly studied is one of the most delicate barometers of the varying conditions of the brain; it is better in the morning after a good nights repose than in the fatigue of the evening; it is better, as all know, in the prime of youth and manhood, than in the decay of old age; it is temporarily imparied and in some cases destroyed by injuring the head or severe disease. It is inevitable that in profound anemia or neurasthenia, temporary deficiency in mental control should be noticed in various ways; inability to add up a column of figures with accustom facility, wandering of the mind from the task before it, reverie and dreams, and irritability, and petulance, under slight provocation in a nature usually calm and kindly. These are some of the symptoms of impairment of memory.

